# Burden of caregiving among caregivers of patients with severe mental illnesses in Benin City, Nigeria

**DOI:** 10.4314/ahs.v22i2.75

**Published:** 2022-06

**Authors:** Caroline E Ofovwe, Samuel O Osasona

**Affiliations:** 1 Department of Mental Health, University of Benin Phone number: +2348033823399. cofovwe@yahoo.com; 2 Department of Mental Health, University of Benin Teaching Hospital, Benin City, Nigeria

**Keywords:** Mental illness, Caregiving, Burden, Nigeria

## Abstract

**Background:**

Severe mental illness exerts a tremendous burden on both the sufferer and caregiver. Such burden has been severally identified as enormous involving psychological, physical and economic challenges.

**Objectives:**

This study examined the prevalence of burden of caregiving among caregivers of patients with severe mental illness; its relationship with the socio-demographic characteristics of the caregivers and patients, and the clinical variables of the patients.

**Methods:**

A cross-sectional descriptive design was employed, and participants included a dyad of 141 consecutive caregivers and patients who met the study inclusion criteria at the psychiatric out-patient clinic of a tertiary hospital. The Zarit Burden Interview was used to collect data from the caregivers; in addition, separate socio-demographic data collection sheets were used to collect data from caregivers and patients accordingly. Version 22 of SPSS was used to analyze the data at a statistically significant level of P< 0.05.

**Results:**

About 37.6% of the caregivers experienced moderate to severe burden of caregiving. Duration of caregiving had a weak positive correlation with burden of care (r=.298, p=.004). While adjusting for other variables, duration of caregiving (OR=1.163, P=.017, 95% CI=1.027–1.317), and poor social support (OR=.438, P=.047, 95% CI=.194-.199) retained independent, statistically significant association with burden of caregiving, explaining about 16% in the variance of burden of caregiving.

**Conclusion:**

There is a need to provide social support for caregivers of persons suffering from mental illness. Subsidization of cost of medication and hospitalization can reduce the burden experienced.

## Introduction

Mental illnesses are generally becoming more prevalent globally and are a leading cause of global burden of disease.^1^,^2^ Schizophrenia, bipolar affective disorder, and major depression are referred to as severe mental illnesses (SMI).^2^ SMI causes substantial burden on caregivers, owing to certain characteristics that distinguish them: First, they, mostly, run a chronic course with intermittent relapses. Researchers have found that 30%–50% of psychiatric patients, especially those with SMI, may experience relapse of symptoms in the first six months, and 50%–70% in the first five years after discharge from the hospital.^3^,^4^ Secondly, they have a debilitating effect on the patients and lead to significant impairment in one or more areas of functioning including loss of productivity^5^, ^6^; thus contributing significantly to “years lived with disability” (YLD).^7^ Third, continuous, long-term care is required by the patients; about 10% of people with a severe mental illness need care in the long term.^8^ Consequent upon these characteristics of SMI, a lot of burden might be placed on the caregiver.

In the past five decades or more, emphasis has been on community-based approach in the care of psychiatric patients, globally^9^, such that, even when hospitalization is required in the acute phase of the illness, they are soon discharged to the community after being stabilized in the therapeutic environment. In Nigeria there is little or no professional follow-up in the community due to grossly inadequate mental health care professionals, resulting in many families having to care for a relative suffering from a mental illness. Yildirim and his colleagues^8^ observed that most patients with a mental illness live with their families, and the relatives become the primary caregivers with attendant increased responsibility and burden.

Caregiver burden has been defined as the emotional, physical, financial demands and responsibilities of an individual's illness that are placed on the family members, friends, or other individuals involved with the individual outside the health care system.^10^ Caregiving is a dynamic process which includes patient and a person who is involved in long term care of the patient.^11^ Although the challenges of caregiving vary across cultures, literature suggests caregivers generally encounter psychological, physical and economic burden which often are enormous.^12^ Caring for those with chronic conditions generally, requires tireless efforts, energy, and empathy, which adversely impact the daily lives of caregivers, and providing for a patient with a major mental illness in particular, portends a negative impact on the quality of life of the caregiver.^13^ Brodaty and Donken^14^ reported that “the strain due to medical costs, missed work and patients' economic dependency are considerable and are linked to both objective and sub-jective burden.”

The scope of caregiving may include a wide range of activities such as assisting the patients in performing their activities of daily living: bathing, cooking, dressing, taking medication and hospital follow-up attendance^13^, ^15^. Substantial body of literature shows that burden of care is associated with many factors which include: duration of illness16; patient's symptoms/diagnosis, social support system, financial resources^17^; age, sex, educational status of the patient and caregiver 18 and severity of illness.^19^ Some studies suggest the number of hospitalizations and the length of illness are most frequently associated with burden in caregiving.^20^,^21^

Across many cultures, especially in Nigeria, caregiving for patients with chronic mental illnesses is often done by family members, friends, non-governmental/voluntary, and religious organizations. However, family members play the most important role in the care of the patients and the prevention of readmission.^22^ Clement and his colleagues^23^ reported that approximately 60%–85% of mentally ill individuals are cared for by their family members. Similarly, it has been observed that family members are responsible for taking care of their mentally ill relatives in developing countries.^13^ Within the family, first degree relatives are more involved in care giving^12^ and may bear more burden of care than distant relatives.

It is important to note that caregivers' burden is a multidimensional concept but commonly categorized in terms of objective and subjective burden^24^. Objective burden includes the outwardly quantifiable demands such as financial cost of illness, time devoted to care, disruptions of family routines and patient's dependence on the caregivers for support for activities of daily living^4^, while subjective burden is defined as the caregivers' attitude or emotional reactions to the caregiving experience^25^. In order to get a broad perspective of the burden of care, it is important to assess both aspects of burden. However, the focus of this study is largely on the objective burden; future study would examine subjective burden.

In view of many empirical evidences in support of enormous emotional, physical and financial burden that caregivers of persons with SMI may suffer,^10^,^12^,^13^ and the need to increase knowledge base in this regard, especially in Nigeria, it is imperative to assess burden of care, and identify caregivers' needs as reported by them. This is an important first step towards prompting appropriate intervention by relevant stakeholders in reducing caregivers' burden, enhancing support, and achieving the goals of treatment for the patients. Although previous studies in Nigeria have made substantial contribution to knowledge base in terms of prevalence and correlates of burden of caregiving, most of them have narrowed their investigations to burden associated with just a diagnostic entity, mostly schizophrenia or dementia, information on the relative influence of the SMI on burden of care is pause. Also, a prominent gap is that the crucial role of social support in caregiving has received little mention. There is a need to contribute to bridging this gap.

To this extent, this study aimed to assess the prevalence of burden of care among caregivers of patients with SMI, and determine the relationship between burden of care and the socio-demographic characteristics of caregivers, and patients, as well as the clinical variables of the patients.

## Methods

### Study setting, design and sample size

This study was conducted at a tertiary hospital in Benin City, Edo state, Nigeria. The foremost referral facility has 850-bed-capacity and receives patients from various parts of the country, but mostly from Edo state and some neighboring South-south, South-west, and South-east states. The psychiatric unit of the mental health department runs outpatiet clinics three times in a week, with an average of 75 patients per week. A cross sectional descriptive design was adopted. Participants included a dyad of 141 psychiatric patients and the caregivers who accompanied them ((a pair of respondents consisting of a patient and his/her caregiver). They were selected consecutively, as they presented for follow-up appointments at the out-patient clinics of the hospital and data collection lasted from September, 2019 till February, 2020.

The sample size (dyad of 141 caregiver/patient) was determined Using single population proportion formula for calculating sample size in a cross sectional study, n= ()13, where a proportion of 16% (prevalence of burden of care, reported in a previous local study)13, a 95% confidence interval, Z=1.96, at 5% margin of error were used.

### Eligibility criteria

Eligibility criteria for patients included: being a psychiatric patient receiving treatment for major depression, bipolar disorder or schizophrenia based on ICD-10 diagnostic criteria, patient not having another known chronic illness, for example diabetes, and being mentally stable enough to give consent and voluntarily participate in the study. Caregivers eligibility criteria included being the/or one of the caregiver(s) of the patient, having being a caregiver of the patient for at least six months, not caring for any other patient with a known chronic illness, being an adult aged 18 years and above, and expression of willingness to voluntarily participate in the study and give consent. Patients and caregivers who did not meet the above criteria respectively, who could not communicate in English or who declined consent were excluded from the study.

### Data collection tool

The data collection tool was partitioned in to two sections described below.

1) Section A: The authors designed separate socio-demographic data collection sheet to collect information from the caregivers and patients respectively, such as age, sex, level of education and so forth. Additional information was obtained from the caregivers regarding the level of social support they get from relevant others, and experience of financial difficulty. It was a self-reported response (based on respondents' subjective satisfaction or experience) in which caregivers were asked to choose which of two options applied to them (“How would you describe the level of social support you get from others ?.........Good/Poor”; Do you experience disturbing fi-nancial difficulty that you could ascribe to caring for this patient ?.........Yes/No). The clinical information of the patients, such as diagnosis, duration of illness and the like, was retrieved from their case notes by a consultant and two resident psychiatrists.

2) Section B: The Zarit Burden Interview^26^: A standard instrument for measuring the level of burden experienced by caregivers of patients with chronic illnesses; it's a 22-item, structured, self-administered questionnaire. Examples of the items are: “Do you feel that your patient asks for more help than he/she needs?”; “Do you feel that because of the time you spend with your patient that you don't have enough time for yourself?” “Do you feel stressed between caring for your patient and trying to meet other responsibilities for your family or home?” Each item requires a response on a 5-point Likert scale: ‘Never’; ‘Rarely’; ‘Sometimes’; ‘Quite frequently’ and ‘Nearly always’; with a score of 0, 1, 2, 3 and 4 respectively. Total scores range from 0 to 88 and the level of burden are graded as: 0 to 20 points = no burden, 21 to 40 points = mild burden, 41 to 60 points = moderate burden and 61 to 88 points = severe burden. According to its original paper, the items had a Cronbach's alpha value of 0.93 and a test- retest reliability of 0.8924; in this sample the Cronbach's alpha was found to be 0.87. The instrument has been used by authors in this local environment to assess burden of care among caregivers of patients with a mental illness^12^, ^13^.

### Procedure and ethical issues

Prior to the commencement of the study, ethical clearance and approval was obtained from the Ethics and Research Committee of the affiliate institution. On each clinic day, potential participants (patient and caregiver) presenting in the clinic consecutively, were approached by one of the principal investigators (a consultant Psychiatrist), and two research assistants who were registrars in psychiatry; the nature and purpose of the study were explained to them in the doctors' consulting rooms, they were informed of their liberty to either participate voluntarily or decline participation, and that there would be no penalty for declining participation, or incentive for participating. Confidentiality was assured and verbal informed consent was obtained from every willing patient and his/her caregiver. Patients who gave consent and met the eligibility criteria underwent a brief clinical mental state assessment by the consultant psychiatrist and the registrars, to establish they were mentally stable enough to participate in the study (absence of gross behavioural abnormality, overt psychotic symptoms, and cognitive impairment), as those symptoms may influence the burden experienced by caregivers. Thereafter, patints and caregivers were given their separate questionnaires which were written in English and self-administered, but participants were told to feel free to seek clarification on any item of the questionnaire as the need arose. Information regarding the clinical variables of the patients was retrieved from their case notes by the consultant psychiatrist and the two assisting resident doctors.

### Data analysis

Data was analyzed using SPSS version 21. Categorical variables were dichotomized as necessary and their frequencies and percentages determined. For the purposes of Chi test, logistic regression, and ease of discussion, burden of care was also dichotomized into “present” and “absent” to represent caregivers with any degree of burden (a Zarit score of 21 and above) and those without burden (a Zarit score of 20 and below) respectively. Chi-square test and correlation coefficient analysis were done to determine caregiver and patient's characteristics that had significant relationship with burden of caregiving. Such significant variables (independent variables) were regressed on caregivers' burden (outcome variable) using binary logistic regression model in order to further confirm the association observed in bivariate analysis. The use of logit transformation was arrived at, based on status consideration (variables that had statistically significant associations at bivariate analysis level), and consideration for better link of relationship between independent and outcome variables in a cross-sectional design. The following assumptions were made and confirmed for the logistic model: a) model fit (the predicted would match the observed), this was confirmed by a non-significant Hosmer and Lemeshow test (Chi2 = 3.312, df=8, P= 0.913); b) sample size would support the modeling using the assumption that 1 independent variable= 10 cases (re-spondents).

## Results

A total of one hundred and forty one (141) caregivers with a mean age of 43.65 13.02 years participated in the study as shown in Table 1. More than half (56.7%) of them were females. One hundred and seventeen (83.0%) had secondary level of education and above./span> A majority (80.1%) were Christians, about two-thirds (66.0%) were employed and 96 (68.1%) were married while 23 (16.3%) were single. The caregivers relationship with patients were either first degree 54 (38.3%), second degree 18 (12.8%), spousal 53 (37.6%) or distant/non biological 16 (11.3%). Sixty-six (47.8%) reported unsatisfactory level of social support, and experience of financial difficulty was reported by 73 (52.5%). Eighty eight (62.4%) of the caregivers had varying degree of burden; 22.7% and 14.9% had moderate and severe burden respectively.

**Table 1:** Socio-demographic and Clinical Characteristics of Caregivers

Variables	Frequency (n=141)	Percentage (%)
**Sex**		
Male	61	43.3
Female	80	56.7
**Level of Education**		
No Formal Education	1	.7
Primary Education	23	16.3
Secondary Education	57	40.4
Tertiary Education	60	42.6
**Religion**		
Christianity	113	80.2
Islam	27	19.1
Others	1	. 7
**Employment Status**		
Employed	93	66.0
Not Employed	48	34.0
**Marital Status**		
Single	23	16.3
Married	96	68.1
Previously Married	22	15.6
**Relationship with Patient**		
First degree	54	38.3
Second degree	18	12.8
Spouse	53	37.6
Distant/Non-biological	16	11.3
**Self-reported Level of Social** Support (n=138)*		
Good support	72	52.2
Poor support	66	47.8
**Self-reported Financial** Difficulty? (n=139)**		
Yes	73	52.5
No	66	47.5
**Degree of Burden**		
No Burden	53	37.6
Mild Burden	35	24.8
Moderate Burden	32	22.7
Severe Burden	21	14.9

* Level of social support item was responded to by 138 of the141 respondents

** Financial difficulty item was responded to by 139 of the 141 respondents

The mean age of the patients was 40.68 5.43; 77 (54.6%) of them were females, 57 (40.4%) were single while 15 (10.6%) were previously married (separated/divorced/widowed), 69 (48.9%) had secondary level of education while 54 (38.3%) had tertiary education and almost two-thirds (63.1%) were unemployed. The highest proportion of the patients (43.3%) was being managed for schizophrenia followed by bipolar affective disorder at 31.9%.

Patients' level of education, employment status, and marital status did not statistically significantly differentiate caregivers who had burden of care from those who did not. However, the experience of burden was most prevalent among caregivers of patients with schizophrenia (73.8%) and least prevalent among caregivers of patients with bipolar affective disorder; the difference was statistically significant (51.1%), (Chi2 = 6.219, df=2 and p = 0.045).

As shown in Table 1V, the differences in social support and financial capability among caregivers who had burden of care versus those without were statistically significant (Chi2 = 8.56, df=1, p = 0.003; and Chi2 = 4.16, df=1 and p = 0.041 respectively). Pearson correlation analysis (table was not shown) revealed a weak but positive and statistically significant correlation between burden of caregiving scores and duration of caregiving (r= .248, p= .004). However burden scores did not have statistically significant correlation with other numerical variables (caregivers' age in years, r=.075, p=.378; caregivers' average monthly income, r=.135, p=.119; monthly medication expenses, r=.124, p=.178; patients' age, r=-.015, p=.863; and patients' average monthly income, r=.082, p=.497).

Although the cross-sectional design of this study did not allow for a predictive conclusion between the independent variables and burden of care, multiple binary logistic regression (Table 5) revealed that duration of caregiving and social support retained statistically significant association with burden of care after adjusting for other variables. Increasing duration of caregiving increased the odds of experiencing burden of care (OR= 1.163, P= .017, CI=1.027–1.317), and the risk of burden is decreased in caregivers with good social support compared to those with poor support [OR=.438, p= 0.047, CI = .194 -.199]. These variables explained about 16.0% of the variance in the burden of care.

**Table 5:** Logistic Regression of independent variables on burden of caregiving

	B (regression co-efficient)	P value	Odds ratio	95% Confidence Interval	
				Lower	Upper
Duration of care	.151	.017	1.163	1.027	1.317
Support(1)	-.826	.047	.438	.194	.990
Financial difficulty(1)	.788	.054	2.198	.987	4.897
Patient diagnosis*		.151			
Schizophrenia(1)	.867	.062	2.380	.957	5.917
Depression(2)	.229	.656	1.258	.458	3.451
Constant	-.498	.418	.608		

* Reference category= Poor social support, Absence of financial difficulty, Bipolar affective disorder. R2 (co-efficient of determination)= 16.0%.

** Statistically significant

## Discussion

This study examined the prevalence of burden of caregiving and the factors that are associated with burden in caregivers of patients with severe mental illness. A majority of the caregivers were females, similar to the findings in some previous studies^9^,^12^,^13^. In most African cultures females take up caregiving roles more than males, this is borne out of societal normative expectation that women have more patience and motherly tendencies as such possess more caring capability than men.

The prevalence of burden of care as found in this study is noteworthy. The overall prevalence of 62.4% (mild to severe) is slightly higher than the rate of 60.4% reported by Inogo and her colleagues^12^ in the same environment, using the same burden assessment instrument and the same scale cut-offs. Their study population was more homogenous, comprising only schizophrenic patients and their caregivers; it would be expected that burden rate in their study would be higher than the rate found in this study since caregivers of schizophrenic patients are generally reported to have higher burdens; Lasebikan and Ayinde^27^, found that the burden of caring for schizophrenic patients is particularly high in Nigeria. It is possible that other factors relted to methodology and caregivers' variables were responsible for the difference in findings. Dada and his colleagues^28^ in their study among a population of caregivers in another Nigerian facility found that the prevalence of moderate level of burden of care was 22.0%, which is comparable to the rate of 22.7% found for moderate burden in this study. However, many previous studies within and outside Nigeria reported rates that were higher than that found in this study^9^, ^13^, ^29^. The relatively stable mental state of our patients as revealed by the mental state examination prior to data collection could be contributory. Besides, variation in findings regarding prevalence of burden of caregiving may be attributable to many factors including study design, study population, sample size and the instrument used to measure burden. Furthermore, literature suggests that outcome may vary between the acute and chronic phase of a severe mental illness^9^. The relatively high burden of caregiving in this study is, perhaps, not surprising; a majority of the caregivers are married and employed, having responsibilities to their families and sources of livelihood. Taking on additional responsibility of caring for a patient with SMI could increase their burden, especially against a backdrop of financial difficulty reported by a majority of them and poor social support reported by almost half of them. This high burden rate underscores the need to incorporate measures that will alleviate the burden of caregivers into the Nigerian mental health care system, thereby improving patients' clinical outcome.

Burden of caregiving was most prevalent among caregivers of patients with schizophrenia compared to caregivers of patients with bipolar disorder and depression, and the difference was statistically significant. Imas and Wandee^16^ similarly reported that caring for a patient with schizophrenia is associated with a significantly higher degree of burden. Besides running a chronic, sometimes non-remitting course, schizophrenia is often associated with negative symptoms which are frequently linked to functional impairment. Research findings report that the effort, energy and empathy required to take care of such patients adversely affect caregivers^13^.

Self-reported poor Social support was found to be significantly associated with burden of caregiving. Chii and his colleagues^30^ reported that perceived social support had an inverse correlation with burden of caregiving. Many other studies reported similar finding^9^,^22^. Social support plays a very crucial role in reducing the burden of caregiving, especially in developing countries like Nigeria where the fulfillment of caregivers' unmet needs depends largely on informal support. The recurrent cost of hospital consultation and medication due to the chronic nature of the illness, particularly for the SMI, marked with a vicious circle of relapse, hospitalization and discharge is enormous. Undoubtedly, this needs social support to stem the tide and provide the needed respite for the caregivers; where this is not available, the caregiver and the patient get thrown into untold difficulties with far reaching implications on their health and wellbeing. Unfortunately, the mental health care system and structure as it is presently in Nigeria, is focued to meet the needs of the patients, with little or no response to the burdensome needs of the caregivers. Thus, for optimum delivery of health care services to persons with SMI the provision of some level of social support to both the patients and caregivers should be addressed to lessen the burden of caregiving.

The finding of significant association between duration of caregiving and burden in this study supports the report by Walke and his colleagues^9^ that increasing duration of caregiving increased the odds of experiencing severe burden among caregivers of mentally ill individuals. Same finding was reported by other authors^11^,^13^,^17^. Expectedly, the longer a caregiver provides care for the patient the longer he/she may have to contend with adverse circumstances such as increasing hospitalization, financial difficulty, physical and psychological distress and so forth.

The significant relationship between self-reported financial difficulty and burden of caregiving in this study is in keeping with previous findings^9^,^31^ and common expectation. Most countries do not provide financial support for patients and their caregivers, thus family members are solely responsible for the financial burden of caring for a mentally ill relative.^13^ Unfortunately, Nigeria is not exempted from this observation. Undoubtedly, caregivers with experience of financial constraint, especially against a backdrop of poor social support network, would have difficulty coping with the financial implications of a chronic SMI, this could be quite burdensome to the caregiver but beyond that, it portends poor adherence to medication on the part of the patient; invariably leading to poor treatment outcome and a worsening of the burden of the caregiver.

Contrary to some previous reports^8^,^9^,^11^,^12^, some seemingly ‘important’ variables such as the patients' level of education, sex, age and employment status did not have a significant relationship with the burden of caregiving in this study.

This study concluded that almost two-thirds of caregivers of patients with SMI experienced varying degrees (mild to severe) of burden of caregiving. Burden had significant association with a diagnosis of schizophrenia, self-reported financial difficulty, poor social support and duration of caregiving.

Due to increasing awareness of the role of caregivers in the management of patients with chronic and severe mental illness and the need to significantly lessen their burden, thereby enhancing the achievement of the goals of treatment in the patients, the investigators recommend that: 1) emphasis in treatment and rehabilitation in psychiatry must be focused not only on the patients as practiced hitherto, but on both the patients and the caregivers; 2) Improvement of Community mental health services will play a crucial role in supporting caregivers and patients within the community; Roick and his colleagues32 reported that utilization of community health services decreases the caregivers' burden as the patients showed significant increase in their health function. 3) There is a need for the establishment of psycho-education and counseling services in mental health settings, a recommendation that is strengthened by Chan and his colleagues' report^33^ that psycho-education programmes play significant role in improving the knowledge and skills necessary to take on the responsibility of caregiving and may ameliorate caregivers' burden.

Although this study revealed the extent of burden among caregivers of patients with SMI and the factors that are associated with the burden, thereby making significant contribution to the body of literature in this regard; it is not without some limitations. The relatively low scale of the research in terms of sample size and institutional coverage, as well as the cross sectional nature of the design, necessitate cautious interpretation of the findings. For future research, a replication of this study on a broader scale and setting, perhaps a longitudinal design is advocated.

## Figures and Tables

**Table 2:** Socio-demographic and Clinical Characteristics of Patients

Variables	Frequency n = 141	Percentage (%)
**Sex**		
Male	64	45.4
Female	77	54.6

**Level of Education**		
No Formal Education	4	2.8
Primary Education	14	9.9
Secondary Education	69	49.0
Tertiary Education	54	38.3

**Employment Status**		
Employed	51	36.2
Not employed	90	63.8

**Marital Status**		
Single	57	40.4
Married	69	49.0
Previously married	15	10.6

**Diagnosis**		
Schizophrenia	61	43.3
Depression	35	24.8
Bipolar affective disorder	45	31.9

**Table 3:** Association between Socio-demographics of patients and caregivers' burden

Variables	Burden		χ^2^	Df	P-value
	No Burden n=53(37.6%)	Presence of burden n=88(62.4%)			
**Level of Education**					
No Formal Education/ Primary Education*	9(50.0)	9(50.0)	2.368	2	0.306
Secondary Education	22(31.9)	47(68.1)			
Tertiary Education	22(40.7)	32(59.3)			
**Employment Status**					
Employed	21(41.2)	30(58.8)	0.559	1	0.455
Not Employer	31(34.8)	58(65.2)			
**Marital Status**					
Single	20(35.1)	37(64.9)	0.531	2	0.767
Married	28(40.6)	41(59.4)			
Previously Married	5(33.3)	10(66.7)			
**Diagnosis**					
Schizophrenia	16(26.2)	45(73.8)	6.219	2	0.045
Depression	15(42.9)	20(57.1)			
Bipolar affective disorder	22(48.9)	23(51.1)			

* The only one respondent that had no formal education was merged with those with primary education (No formal education/primary education) for the purpose of the test of association.

**Table 4:** Association between Socio-demographics of caregivers and burden of caregiving

Variables	Burden		χ^2^	Df	P-value
	No Burden n=53 (37.6%)	Presence of burden n=88 (62.4%)			
**Sex**					
Male	26(42.6)	35(57.4)	1.161	1	0.281
Female	27(33.8)	53(66.3)			
					
**Level of Education**					
No Formal Education/ Primary Education*	8(33.3)	16(66.7)	2.643	2	0.266
Secondary Education	26(45.6)	31(54.4)			
Tertiary Education	19(31.7)	41(68.3)			
					
**Religion**					
Christianity	45(39.8)	68(60.2)	3.466	2	0.177
Islam	7(25.9)	20(74.1)			
Others	1(100.0)	0(0.0)			
					
**Employment Status**					
Employed	33(35.5)	60(64.5)	0.516	1	0.473
Not Employed	20(41.7)	28(58.3)			
					
**Marital Status**					
Single	7(30.4)	16(69.6)	3.646	2	0.162
Married	41(42.7)	55(57.3)			
Previously Married	5(22.7)	17(77.3)			
					
**Relationship with** **Patient**					
First degree	16(29.6)	38(70.4)	4.319	3	0.229
Second degree	6(33.3)	12(66.7)			
Spouse	22(41.5)	31(58.5)			
Distant/non-biological	9(56.3)	7(43.8)			
					
**Level of Social** **Support**					
Good support	36(50.0)	36(50.0)	8.555	1	0.003
Poor support	17(25.8)	49(74.2)			
					
**Financial Difficulty?**					
Yes	22(30.1)	51(69.9)	4.163	1	0.041
No	31(47.0)	35(53.0)			

* Respondents with no formal education were merged with those of primary education.

## References

[1] Mathrs C, Fat D M, Boerma JT (2000). The global burden of disease: 2004 update.

[2] World Health Organization (WHO) Mental Disorders fact sheet. Reviewed 2016.

[3] Chang Y C, Chou F C (2015). Effects of Home Visit Intervention on Re-hospitalization Rates in Psychiatric Patients. Community Mental Health J.

[4] Awad A G, Voruganti L N P (2012). The Burden of Schizophrenia on Caregivers. Pharmaco Economics.

[5] Knapp M (1997). Costs of Schizophrenia. The British Journal of Psychiatry.

[6] Adeosun I I (2013). Correlates of Caregivers burden among Family Members of Patients with Schizophrenia in Lagos, Nigeria. Schizophrenia Research and Treatment.

[7] Geriani D, Sarithry K S, Shivakumar S, Kanchan T (2015). Burden of Care on Caregivers of Schizophrenic Patients: A Correlation to Personality and Coping. J Clin Diagn Reb.

[8] Yildirim S, Yalciner N, Guler C (2017). Caregiver Burden in Chronic Mental Illness: A Systematic Review. Journal of Psychiatric Nursing.

[9] Walke S C, Chandrasekaran V, Mayya S S (2018). Caregivers Burden among Caregivers of Mentally Ill Individuals and their Coping Mechanism. J Neurosci Rural Pract.

[10] Ravi S, Goud BR, Archana M, Pius T M, Pal R, John V (2013). Burden among caregivers of Mentally Ill Patients: A Rural Community-based Study. Int J Reb Dev Health.

[11] Pratima B, Bhatia M S, Jena SPK (2011). Caregiver Burden in Severe Mental Illness. Delhi Psychiatric Journal.

[12] Inogo C F, Olotu S O, James B O, Nna E O (2017). Burden of Care amongst Caregivers Who are First Degree Relatives of Patients with Schizophrenia. The Pan African Medical Journal.

[13] Ajibade B L, Ajao O O, Fabiyi B, Olabisi O I, Akinpelu A O (2016). Burden experienced by family caregivers of patients with mental disorders at selected hospitals in Ekiti State, Nigeria. Int J of Health and Psychology Research.

[14] Brodaty H, Donkin M (2009). Family Caregivers of People with Dementia. Dialogues C in Neurosic.

[15] Grover S (2015). Chakrabartis. Coping among the Caregivers of patients with schizophrenia. Ind Psychiatry J.

[16] Imas RS, Wandee S (2011). Review: Burden on Family Caregivers Caring for Patients with Schizophrenia and its Related Factors. Nurse Media Journal of Nursing.

[17] Alejandra C U, Jose G M, Marta F G, Claudia P S, David R A, Alejandro C P (2011). Attitude and Burden in Relatives of Patients with Schizophrania in a Middle Income Country. BMC Farm Pract.

[18] Zegwaard M I, Aartsen M J, Grypdonck M H F, Cuijpers P (2013). Differences in Impact of Long Term Caregiving for Mentally Ill Older Adults on the Daily Life of Informal Caregivers: A Qualitative Study. BMC Psychiatry.

[19] Karlikay G, Yukse G, Varlibas F, Tireli H (2005). Caregiver Burden in Dementia: A Study in the Turkish Population. Internet J Neurol.

[20] Grad J, Sainsbury P (1968). The effect that Patients have on their family in Community Care and Control Psychiatric Services: A Two Years Follow up. Br J Psychiatry.

[21] Hoeinig J, Hamilton M W (1996). The Schizophrenic Patient in the Community and his Effect on the Household. Int J Soc Psychiatry.

[22] Akbari M, Alavi M, Irajpour A, Maghsondi J (2018). Challenges of Family Caregivers of Patients with Mental Disorders in Iran: A Narrative Review. Iran J Nursing Midwifery Res.

[23] Clement S, Gerber D, McGuire S L (1995). Comprehensive Community Health Nursing: Family Aggregate and Community Practice.

[24] Schene AH, van Wijngaarden B, Koeter MW (1998). Family caregiving in Schizohrenia: domains and distress. Schizophrenia Bulletin.

[25] Ampalam P, Gunturu S, Padma V (2012). A comparative study of caregiving burden in psychiatric illness and chronic medical illness. Indian J Psychiatry.

[26] Zarit S H, Reever K E, Back-Peterson J (1980). Relatives of impaired elderly: Correlates of feelings of burden. Gerontologist.

[27] Lasebikan VO, Ayinde OO (2013). Family burden in caregivers of schizophrenia patients: prevalence and socio-demographic correlates. Indian J Psychol Med.

[28] Dada MU, Okewole NO, Ogun OC, Bello-Mojeed MA (2011). Factors associated with caregiver burden in a child and adolescent psychiatric facility in Lagos, Nigeria: a descriptive cross sectional study. BMC Pediatr.

[29] Oshodi YO, Adeyemi JD, Aina OF, Suleiman TF, Erinfolami AR, Umeh C (2012). Burden and psychological effects: Caregiver experiences in a psychiatric outpatient unit in Lagos, Nigeria. African Journal of sychiatry.

[30] Chii JC, Hsing-Yi C, Pin C, Hsiu HW (2009). Social support and caregiving circumstances as predictors of caregiver burden in Taiwan. Archives of Gerontology and Geriatrics.

[31] Sen BK, Luo N, Ng WY, Lim J, Chionh HL, Goh J (2010). Validity and reliability of the Zarit Burden Interview in assessing caregiving burden. Ann Acad Med Singapore.

[32] Roick C, Heider D, Bebbinton PE, Angermeyer MC, Azorin JM, Brugha TS, Kernfield A (2007). Burden on caregivers of peole with schizophrenia: Comparison between Germany and Britain. The British Journal of Psychiatry.

[33] Chan S, Yip B, Tso S, Cheng BS, Tam W (2009). Evaluation of psychoeducation programmes for Chinese clients with schizophrenia and their family caregivers. Patient Education and Counselling.

